# Random versus Game Trail-Based Camera Trap Placement Strategy for Monitoring Terrestrial Mammal Communities

**DOI:** 10.1371/journal.pone.0126373

**Published:** 2015-05-07

**Authors:** Jeremy J. Cusack, Amy J. Dickman, J. Marcus Rowcliffe, Chris Carbone, David W. Macdonald, Tim Coulson

**Affiliations:** 1 Department of Zoology, University of Oxford, Oxford, United Kingdom; 2 Institute of Zoology, Zoological Society of London, London, United Kingdom; 3 Department of Zoology, The Recanati-Kaplan Centre, Wildlife Conservation Research Unit, University of Oxford, Oxford, United Kingdom; University of Colorado, UNITED STATES

## Abstract

Camera trap surveys exclusively targeting features of the landscape that increase the probability of photographing one or several focal species are commonly used to draw inferences on the richness, composition and structure of entire mammal communities. However, these studies ignore expected biases in species detection arising from sampling only a limited set of potential habitat features. In this study, we test the influence of camera trap placement strategy on community-level inferences by carrying out two spatially and temporally concurrent surveys of medium to large terrestrial mammal species within Tanzania’s Ruaha National Park, employing either strictly game trail-based or strictly random camera placements. We compared the richness, composition and structure of the two observed communities, and evaluated what makes a species significantly more likely to be caught at trail placements. Observed communities differed marginally in their richness and composition, although differences were more noticeable during the wet season and for low levels of sampling effort. Lognormal models provided the best fit to rank abundance distributions describing the structure of all observed communities, regardless of survey type or season. Despite this, carnivore species were more likely to be detected at trail placements relative to random ones during the dry season, as were larger bodied species during the wet season. Our findings suggest that, given adequate sampling effort (> 1400 camera trap nights), placement strategy is unlikely to affect inferences made at the community level. However, surveys should consider more carefully their choice of placement strategy when targeting specific taxonomic or trophic groups.

## Introduction

Camera trap surveys are used worldwide to inventory and monitor terrestrial mammal communities [1–3]. Common outputs from these studies include an assessment of the number of species present (community richness), their identity (community composition) and the distribution of their absolute or relative abundances (community structure), all of which may be used to guide conservation actions [4]. As with most sampling methods that only survey a small fraction of often vast and heterogeneous landscapes [5,6], choice of an appropriate camera trap survey design is key to obtaining unbiased estimates of these measures [7,8], especially when space use is highly variable across species [9]. In particular, the strategy used to determine the exact location of cameras down to a few meters (hereafter, placement strategy) can have a considerable influence on species detection probability [10], thereby affecting inferences made at the community level.

Non-baited camera trap placement strategies can be broadly classified into two types [11]. Non-random placements target features of the landscape—such as game trails, roads, water points and salt licks—that increase the probability of photographing one or several target species. These are typically used in the context of mark-recapture [12–14] and occupancy [15,16] studies directed at rare or elusive species. Survey designs in which the approximate location of camera traps across the landscape are chosen randomly, but where exact camera placement is determined by specific features of those locations (e.g. game trails), also fall into this category [1,2]. In contrast, random camera placements are determined a priori by precise geographical coordinates and ignore nearby features that may increase capture probability, meaning that such features are sampled in proportion to their occurrence in the landscape [17]. In theory, not only should such randomisation allow a wider variety of landscape features to be sampled, but it should also avoid sampling bias when assessing the presence or relative abundance of multiple sympatric species, particularly when the latter show contrasting space use patterns.

In practice, however, non-random camera placements are still commonly used to draw inferences on the richness, composition and structure of mammal communities, regardless of potential biases arising from sampling only a limited set of habitat features [1–3,18–20]. Recent studies have highlighted how the use of game trails (i.e. paths created by animals—hereafter, trails), in particular, can vary across species [21,22], and how this may affect camera trap survey design [9]. For instance, using strictly random camera placements defined as locations within 5 m of a pre-determined GPS point, Wearn et al. [10] recently obtained a significantly higher detection probability for the endangered and poorly known Bornean bay cat (*Pardofelis badia*) relative to previous camera trap studies that used strictly non-random placements. In the Neotropics, Di Bitetti et al. [23] and Blake & Mosquera [24] reached contrasting conclusions on the influence of trail and off-trail sampling in the context of mammal community surveys. Although these studies provide interesting insights into animal space use, they are of limited use to camera trap survey design since a strategy of placing cameras exclusively off-trails is extremely rare in practice.

With the proliferation of large-scale camera trap studies that aim to make inferences at the community level, it has now become important to quantify potential biases arising from the use of non-random camera placements. Past assessments carried out in tropical forest habitats have tended to compare random and trail-based placements that were in different spatial locations, thereby introducing the possibility that the observed differences could have been due to habitat heterogeneity [9,10]. Here, we implement a paired design to survey medium to large terrestrial mammal species within Tanzania’s Ruaha National Park, with strictly game trail-based and strictly random camera placements located within 50 m of each other. We first assess whether the observed communities differ in richness and composition. We then compare the structure of observed communities in terms of their rank abundance distributions (RADs). Finally, we evaluate what makes a species significantly more likely to be caught at trail placements.

## Methods

### Ethics statement

Data collection was based on the use of remotely set camera traps, a non-invasive method that does not involve contact with the study species, nor interfere with their natural behaviour. Fieldwork was carried out under research permit no. 2013-285-NA-2013-105 to JJC, issued by the Tanzanian Commission for Research and Technology (COSTECH).

### Study area

**Fig 1:**
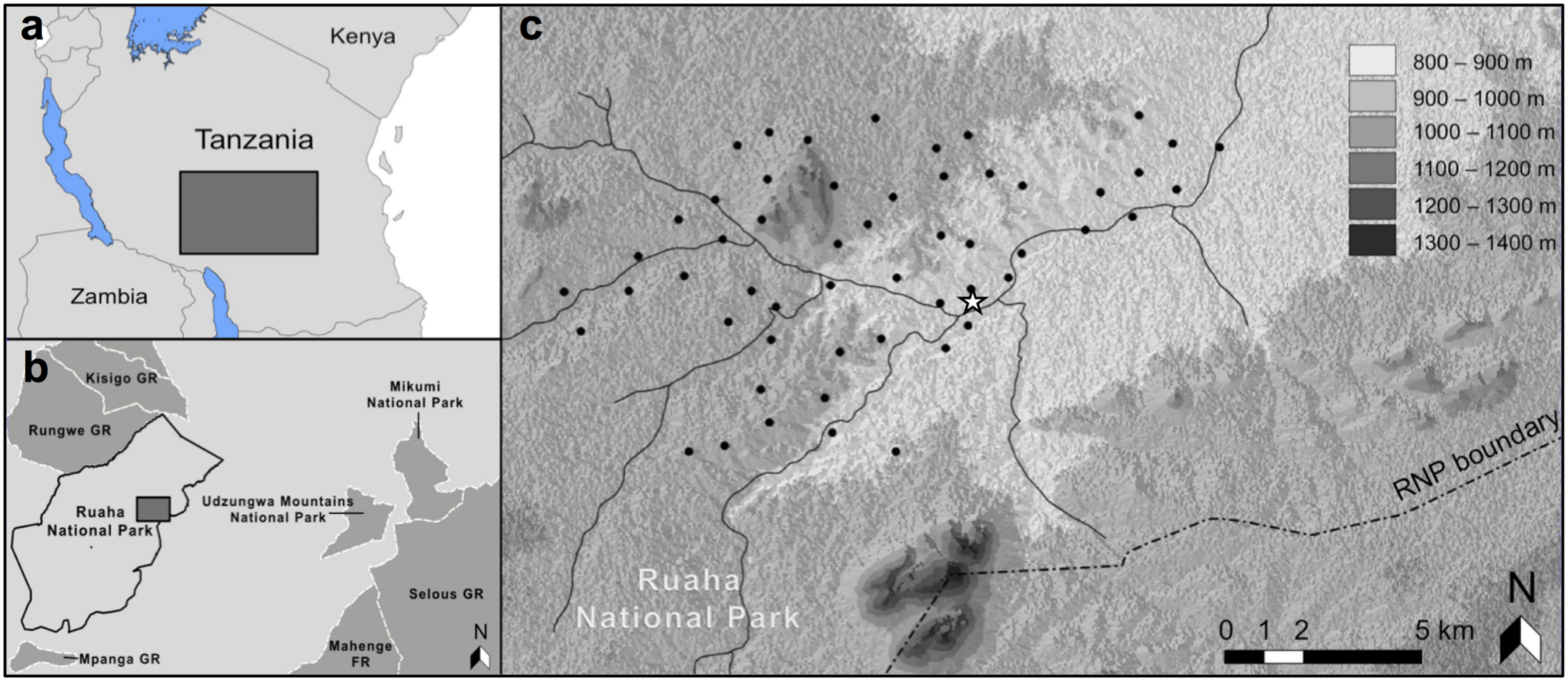
Location of random camera placements within Ruaha National Park, southern Tanzania. Insets (a) and (b) show the location of Ruaha National Park (RNP) in Southern Tanzania and that of the study area on the eastern side of RNP. In (c), solid black lines symbolise the river system present around the study area and the white star marks the location of RNP headquarters. Trail-based camera placements were chosen within 50 m of the random placements (back dots).

The study area is situated on the eastern side of Ruaha National Park (RNP) in southern Tanzania between 7°35’- 7°42’ S and 34°50’- 34°59’ E (Fig 1). RNP is Tanzania’s largest National Park, encompassing an area of 20 226 km^2^, and supports a diverse community of mammal species, including a full guild of large carnivores [25]. In contrast to protected areas in northern Tanzania, the Ruaha ecosystem remains largely unstudied. Roads and tourist facilities are concentrated around the Great Ruaha River, which runs along the south-eastern boundary of the Park. Our study focuses on an area of approximately 100 km^2^ situated close to the Park headquarters (Fig 1).

The climate of RNP is semi-arid to arid, with rainfall peaks occurring from December to January and March to April, and an average annual rainfall of 500 mm [26]. Altitude across the landscape ranges from 696 to 2171 m asl. The vegetation cover is a mosaic of typical East African semi-arid savannah and northerly Zambesian miombo woodland, including *Acacia*, *Combretum* and *Comniphora* species [27]. The Great Ruaha River is the main water supply in the study area, providing a key resource for most wildlife during the dry season from June to November.

### Camera trap surveys

The study area was first divided into 2-km^2^ grid cells in Quantum GIS [28]. Fifty-four adjacent cells were then selected based on ease of access in the field to make up a continuous camera trap array (Fig 1). The location of the random camera placement within each cell was chosen randomly and located in the field using a handheld GPS device (Garmin Etrex 10, Garmin International, Inc., Olathe, Kansas, USA). Placement was on the closest tree within a radius of 5 m (or pole if no tree was present) and oriented so as to offer a reasonably uncluttered view. We then identified a clear, natural game trail within a maximum of 50 m of the random placement on which to position the trail-based camera. For the purpose of this study, we defined game trails as continuous, grassless routes through the habitat measuring at least 1 m in width and showing clear evidence of current usage by wild animals (e.g. presence of fresh droppings, spoor, or recently flattened grass either side of the trail). Taken together, the random and trail-based cameras within a given cell formed a sampling pair. It is important to note that, whereas trail-based cameras could never be placed off-trail, random cameras could be placed on a trail if the latter occurred within the 5 m radius defined above. In our case, 16.7% of the random cameras were positioned on trails.

We took appropriate steps to ensure that no factors other than placement strategy could influence the detection or non-detection of a species. Both cameras within a sampling pair were of the same model (Reconyx HC500, Reconyx, Inc., Holmen, Wisconsin, USA), used the same SD cards (SanDisk 8 GB class 10, SanDisk, Milpitas, California, USA), and all cameras were placed on trees or poles at a height of 0.3 m off the ground. Trail-based cameras were positioned between 3 and 5 meters away from, and at an angle to, the trail to ensure adequate detection of faster moving animals. All cameras were set to take five successive photos per trigger with no delay between consecutive triggers. Date and time were automatically stamped onto each image. Vegetation was cleared for a few meters in front of each camera but not otherwise disturbed. Altitude across camera locations was recorded using a handheld GPS device and ranged from 801 to 956 m (mean = 965.2 m, SD = 40.2 m). The relatively short spacing between paired cameras (≤ 50 m) ensured they sampled the same habitat type.

Sampling periods consisted of 8 successive weeks in both the dry (19^th^ September 2013–18^th^ November 2013) and wet season (21^st^ December 2013–21^st^ February 2014). Cameras were checked once after four weeks of sampling to change batteries and download memory cards.

### Data analysis

Photographed animals were identified down to species level using published guides [29]. Photographic events of the same species were judged to be temporally independent if they were separated by more than one hour [30]. For each camera location (random and trail-based), we computed species-specific relative abundance indices (RAIs) as the number of independent events divided by the number of days the camera was active, and multiplied by 100 (i.e. events per 100 camera trap days; [31,32]). RAIs were also calculated at the survey-level by considering the sum of all events and camera days across a particular survey. Our analyses consider medium to large terrestrial mammals weighing more than 0.5 kg.

Sample-based species accumulation curves were compared using 95% confidence intervals drawn from 200 randomisations performed with replacement. We followed the method of Colwell et al. [33] and computed confidence intervals based on the unconditional variance. For both seasons, curves were compared at the value denoting the lowest effort between the two types of survey [34]. We used the Jaccard dissimilarity index [35] to quantify compositional differences between the observed communities. To compare community structure, we only considered species that were detected in both surveys and whose number of independent events recorded overall was equal or greater than an arbitrary value of five. We fitted null, pre-emption, lognormal, Zipf and Zipf-Mandelbrot models to rank abundance distributions (see [36] for details) constructed from the survey-level RAIs of selected species. Model selection was based on the deviance criterion defined as the minimisation of sum of squares of deviations from predicted and observed values [36]. Although the fitting of models to RADs is an intuitive way of representing and comparing community structures, it does not in itself provide information on the relative rank each species occupies within the observed communities (i.e. rank shifts). In order to assess the latter, we calculated the mean absolute rank shift (MARS) from the random to the trail-based survey using the following formula (see [37], Eq 1):

$$\text{MARS }=\;\sum_{i=1}^{n}\left(\left|R_{i,trail}-R_{i,random}\right|\right)/n$$ (1)

where *n* is the number of species considered, and *R*_*i*_ is the relative rank of species *i* on *random* and *trail*-based surveys. We tested the hypothesis that the MARS was not significantly different from zero using a Wilcoxon signed rank test.

For the same reduced set of species, we also compared RAIs obtained at random and trail-based placements using pairwise Wilcoxon signed rank tests. The latter tested the null hypothesis that the distribution of pairwise differences (trapping rates at random placements minus those at trail placements) was symmetric about zero. The mean of the resulting normal distribution—termed the location shift—and the associated 95% confidence intervals were used to assess the level of significance (α = 0.05) relative to zero. Species for which the RAI at trail placements was found to be significantly higher than that at random placements were given a score of 1 whilst others were given a score of 0. We modelled the resulting binary variable as a function of trophic category (carnivore, herbivore, insectivore and omnivore—classification based on [38]), log body mass (taken from [39]) and social behaviour (solitary/social) using a generalised linear model with binomial errors and a logit link function (S1 Table). We also considered the interaction between trophic category and log body mass. Model selection was carried out using Akaike’s Information Criteria (AIC; [40]), with subsequent inferences based on the model with the lowest AIC value [41].

All analyses were carried out in R version 3.0.3 [42]. Species accumulation curves were plotted using the package iNEXT [43] and analyses of community composition and structure were carried out in package vegan [44].

## Results

### Comparison of community richness, composition and structure

Overall, we detected a total of 41 medium-to-large terrestrial mammal species from 10 567 camera-trap days accumulated across seasons and survey types (Table 1). The number of false triggers was high for both types of survey, representing 45.1% and 42.6% of all triggers taken by the random and trail-based surveys. False triggers were associated with camera locations in grassland areas, where the increased occurrence of swaying grass caused cameras to trigger even in the absence of any animal. The smallest species we consider in our analysis is the slender mongoose (*Herpestes sanguinea*—0.6 kg on average) and the largest, the African elephant (*Loxodonta africana*—3940 kg). Human activities were recorded once at two trail-based camera locations (off-roading vehicles).

**Table 1:** List of medium to large terrestrial mammal species (> 0.5 kg) camera trapped in Ruaha National Park, Tanzania.

			Independent events				Survey-level RAI			
			Dry season		Wet season		Dry season		Wet season	
Taxonomic group	Latin name	Common name	Random	Trail-based	Random	Trail-based	Random	Trail-based	Random	Trail-based
Primata	*Cercopithecus pygerythrus*	Vervet monkey	9	14	8	17	0.32	0.50	0.34	0.66
	*Papio cynocephalus*	Yellow baboon	95	120	67	102	3.34	4.26	2.85	3.99
Carnivora	*Canis mesomelas*	Black-backed jackal	44	133	30	65	1.55	4.72	1.28	2.54
	*Octocyon megalotis*	Bat-eared fox	44	28	26	15	1.55	0.99	1.11	0.59
	*Lycaon pictus*	African wild dog	-	-	-	2	0.00	0.00	0.00	0.08
	*Mellivora capensis*	Honey badger	3	9	5	4	0.11	0.32	0.21	0.16
	*Mungos mungo*	Banded mongoose	3	19	4	7	0.11	0.67	0.17	0.27
	*Bdeogale crassicauda*	Bushy-tailed mongoose	2	2	-	1	0.07	0.07	0.00	0.04
	*Herpestes sanguinea*	Slender mongoose	9	15	2	7	0.32	0.53	0.09	0.27
	*Ichneumia albicauda*	White-tailed mongoose	35	59	11	26	1.23	2.09	0.47	1.02
	*Crocuta crocuta*	Spotted hyena	99	211	62	165	3.48	7.48	2.64	6.45
	*Proteles cristata*	Aardwolf	19	78	28	48	0.67	2.77	1.19	1.88
	*Genetta genetta*	Common genet	18	60	15	26	0.63	2.13	0.64	1.02
	*Genetta tigrina*	Blotched genet	1	-	-	4	0.04	0.00	0.00	0.16
	*Civettictis civetta*	African civet	15	35	4	11	0.53	1.24	0.17	0.43
	*Felis sylvestris*	Wild cat	2	24	6	2	0.07	0.85	0.26	0.08
	*Felis serval*	Serval	4	21	17	18	0.14	0.74	0.72	0.70
	*Felis caracal*	Caracal	1	1	-	2	0.04	0.04	0.00	0.08
	*Acinonyx jubatus*	Cheetah	-	2	2	1	0.00	0.07	0.09	0.04
	*Panthera pardus*	Leopard	12	54	10	36	0.42	1.91	0.43	1.41
	*Panthera leo*	Lion	12	44	8	40	0.42	1.56	0.34	1.56
Pholidota	*Smutsia temminckii*	Ground pangolin	1	-	1	-	0.04	0.00	0.04	0.00
Rodentia	*Hystrix cristata*	Crested porcupine	1	23	3	20	0.04	0.82	0.13	0.78
Ungulata	*Orycteropus afer*	Aardvark	13	13	5	13	0.46	0.46	0.21	0.51
	*Loxodonta africana*	African elephant	354	428	1146	1671	12.45	15.18	48.83	65.35
	*Equus quagga*	Common zebra	162	172	144	168	5.70	6.10	6.14	6.57
	*Hippopotamus amphibious*	Hippopotamus	42	123	66	201	1.48	4.36	2.81	7.86
	*Potamochoerus larvatus*	Bush pig	1	3	-	4	0.04	0.11	0.00	0.16
	*Phacochoerus africanus*	Warthog	34	36	68	84	1.20	1.28	2.90	3.29
	*Giraffa camelopardalis*	Giraffe	229	390	110	165	8.05	13.83	4.69	6.45
	*Syncerus caffer*	African buffalo	10	12	-	-	0.35	0.43	0.00	0.00
	*Tragelaphus scriptus*	Bushbuck	6	5	1	1	0.21	0.18	0.04	0.04
	*Tragelaphus imberbis*	Lesser kudu	20	25	44	28	0.70	0.89	1.87	1.10
	*Tragelaphus strepsiceros*	Greater kudu	170	215	88	112	5.98	7.62	3.75	4.38
	*Taurotragus oryx*	Eland	4	3	5	9	0.14	0.11	0.21	0.35
	*Sylvicapra grimmia*	Bush duiker	60	41	14	36	2.11	1.45	0.60	1.41
	*Oreotragus oreotragus*	Klipspringer	-	2	-	1	0.00	0.07	0.00	0.04
	*Madoqua kirkii*	Kirk's dikdik	52	139	69	146	1.83	4.93	2.94	5.71
	*Gazella granti*	Grant's gazelle	1	1	5	4	0.04	0.04	0.21	0.16
	*Aepyceros melampus*	Impala	1173	1929	996	1103	41.26	68.40	42.44	43.14
	*Kobus ellipsiprymnus*	Waterbuck	22	23	3	6	0.77	0.82	0.13	0.23

The number of independent photographic events and the survey-level relative abundance index (RAI) are given for each season (dry and wet) and type of survey (random versus trail-based placement).

**Fig 2:**
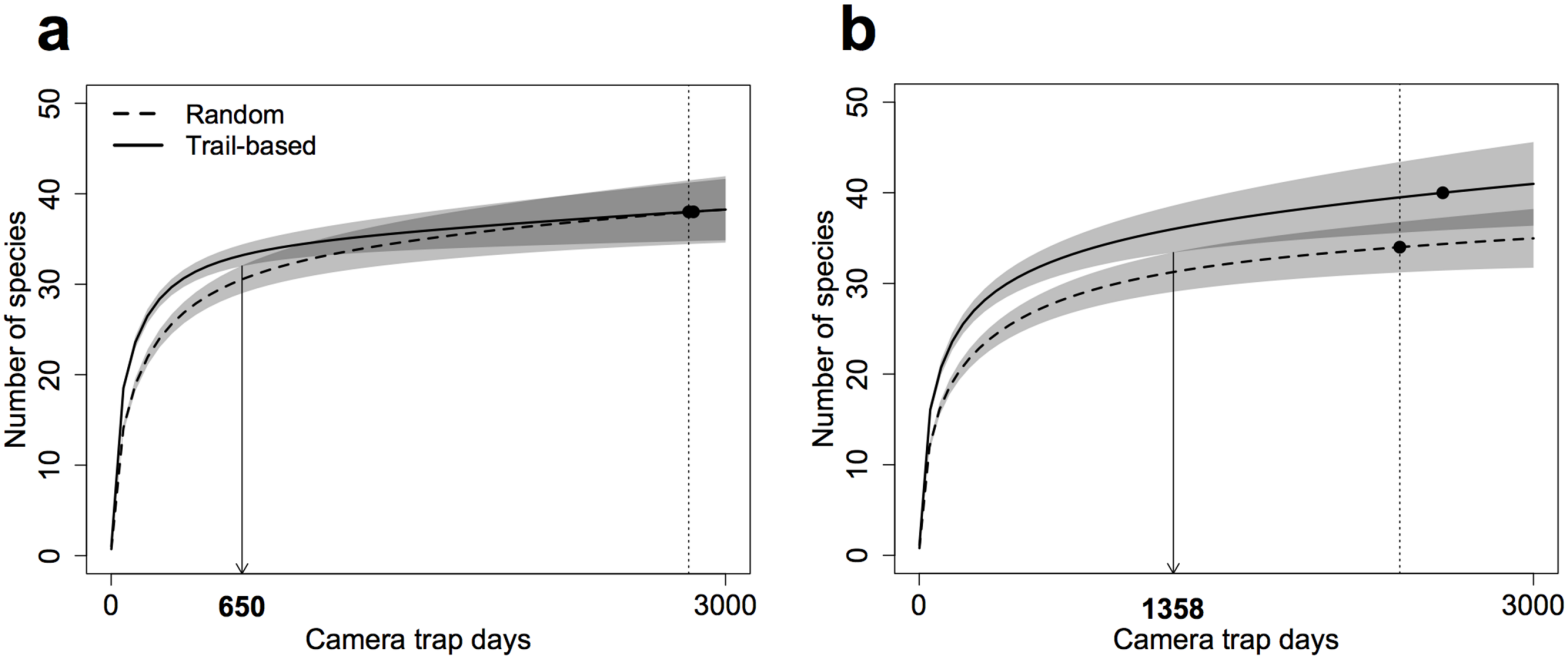
Sample-based species accumulation curves describing the medium to large mammal community richness in the study area during the (a) dry and (b) wet season. Shaded polygons denote the 95% confidence intervals drawn from 200 randomisations performed with replacement and based on the unconditional variance. Confidence interval overlap is shown in a darker shade of grey. For each season, curves were compared at sampling efforts symbolised by the dotted vertical lines. Downward pointing arrows and bold numbers on the x-axis mark the level of effort at which the richness of observed communities could not be considered as significantly different.

We compared sample-based species accumulation curves at sampling efforts of 2820 and 2347 camera trap days in the dry and wet season, respectively (Fig 2). Observed species richness did not differ between the two survey types during the dry season (*S*_*obs*_ = 38 in both cases). However, a minimum of 650 camera trap days was required to obtain communities that did not differ significantly in their richness. The trail placement survey detected more species during the wet season (*S*_*obs*_ = 40) than the random placement survey (*S*_*obs*_ = 34), although confidence intervals overlapped marginally at the point of comparison. In contrast to the dry season, at least 1358 camera trap days were required during the wet season to obtain communities that were not significantly different in their richness. In both seasons, the trail-based placement survey reached higher community richness for lower levels of sampling effort (Fig 2).

Compositional dissimilarity between observed communities was greatest during the wet season when 19.1% of the species detected overall were detected by only one of the surveys. During the dry season, communities were more similar, with only 9.8% of the species being detected by only one survey type. Importantly, community dissimilarity was primarily due to the detection/non-detection of relatively rare (e.g. the African wild dog and the ground pangolin) or habitat-specific species (e.g. the Klipspringer) (Table 1).

**Fig 3:**
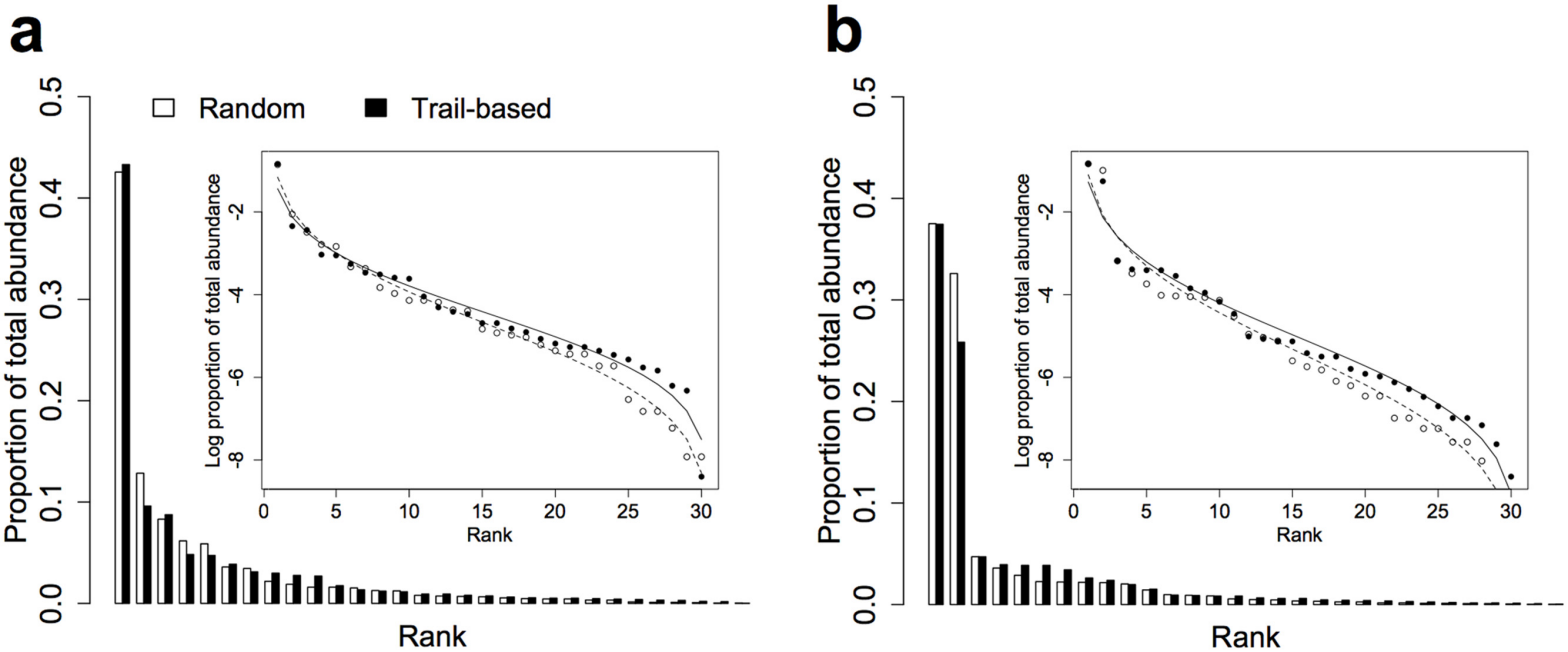
Rank abundance distributions and lognormal model fits (inset plots) for the (a) dry and (b) wet season. A set of 30 species was considered in order to facilitate comparison, with ranks based on RAIs measured at the survey level. Species are ranked from 1 to 30 on the x-axis according to decreasing proportion of total abundance.

Thirty species were considered in the analysis of community structure, including 9 carnivores, 12 herbivores, 4 insectivores and 5 omnivores. Based on the deviance criteria, the lognormal distribution provided the best fit for observed RADs resulting from both types of survey as well as for both seasons (Table 2; Fig 3), thus indicating similar overall community structures. Despite these similarities in overall community structure, the MARS for both seasons was significantly different from 0 (Dry: V = 351, *N* = 30, *P* < 0.001; Wet: V = 253, *N* = 30, *P* < 0.001), indicating that species occupied different ranks within the observed communities.

**Table 2:** Deviance criteria for the five types of model fitted to rank abundance distributions.

Placement	Season	Deviance criterion by model^a^				
		Null	Pre-emption	Lognormal	Zipf	Zipf-Mandelbrot
Random	Dry	23.8	4.9	1.1	8.0	4.6
	Wet	38.6	7.1	3.4	7.4	4.2
Trail	Dry	14.2	6.8	2.2	5.2	4.2
	Wet	23.2	6.2	2.3	4.4	2.8

^a^The deviance criterion is defined as the minimisation of sum of squares of deviations from predicted and observed values.

### Species-level determinants of trail use

In both the dry and the wet season, no species exhibited significantly higher RAIs at random camera placements relative to trail-based ones (S1, S2 and S3 Tables). We therefore only assessed the determinants of species being caught significantly more often at trail placements, which numbered 16 and 12 out of 30 during the dry and wet season, respectively.

**Fig 4:**
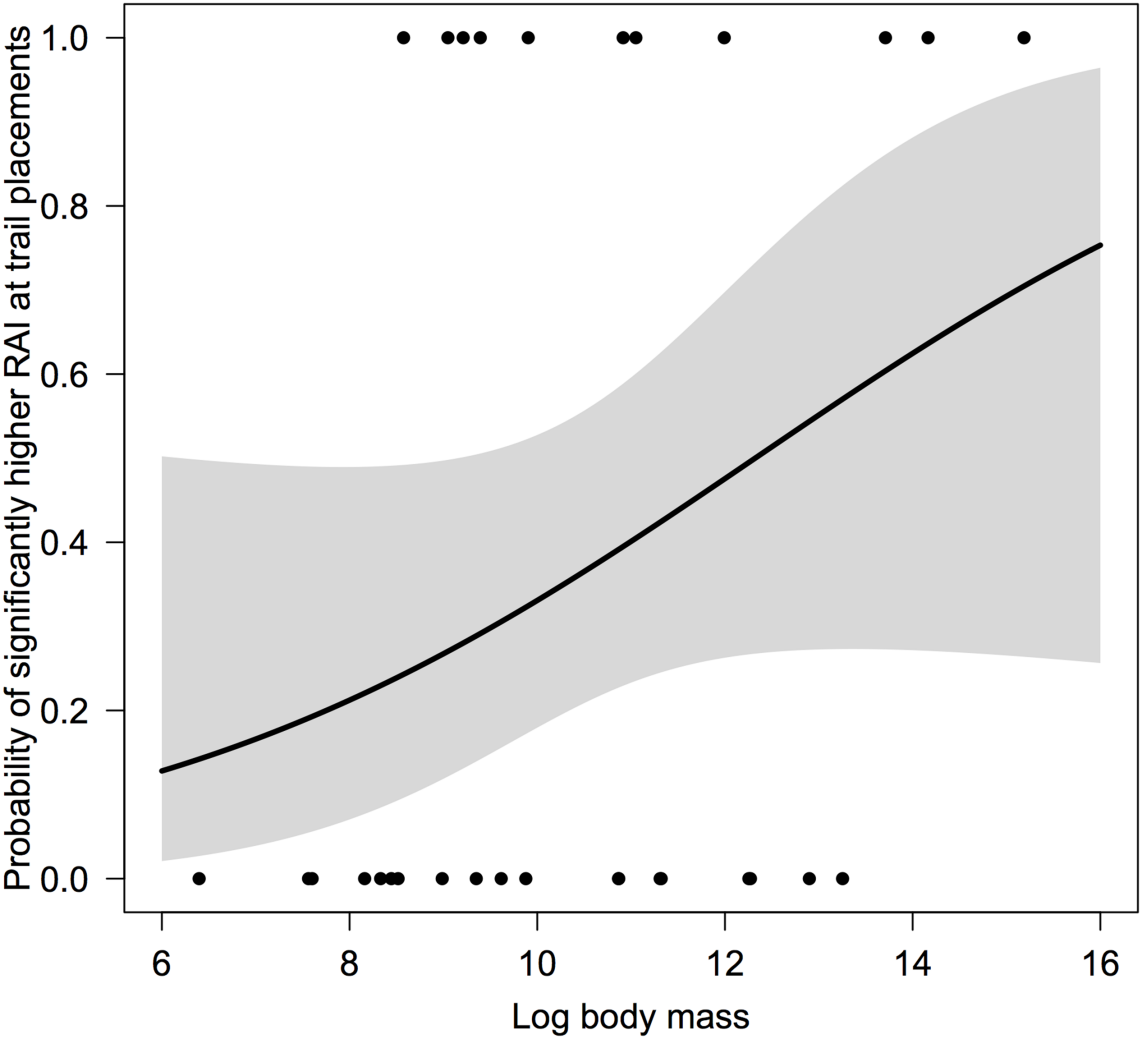
Influence of log body mass on the probability for a species to be caught significantly more often at trail placements during the wet season. The shaded polygon represents the 95% confidence interval surrounding the regression line and black dots represent the species-specific binomial responses used in the generalised linear model.

During the dry season, trophic category was found to be the only predictor retained in the model with the lowest AIC value. Carnivore species, in particular, showed a significantly higher probability of being caught at trail placements (z = 1.96, P = 0.035), with a back-transformed estimate of 0.89 (95% CIs: 0.50–0.98). It is worth noting that the slender mongoose (*H*. *sanguinea*) was the only carnivore species for which RAIs at trail and random placements were not significantly different. In contrast, herbivores were more likely to show equal RAIs across paired trail and random placements (z = -2.54, P = 0.011). The probability for a species within this trophic category to have a higher RAI at trail placements was 0.25 (95% CIs: 0.08–0.55). The hippopotamus (*Hippopotamus amphibious*), giraffe (*Giraffa camelopardalis*) and dikdik (*Madoqua kirkii*) were the only herbivore species to show significantly higher RAIs at trail placements. The probabilities of insectivore and omnivore species having higher RAIs at trail placements were intermediate between those of carnivores and herbivores. The back-transformed probability for an insectivore species of having a higher RAI at trail placements was 0.5 (95% CIs: 0.12–0.88), whilst that for an omnivore species was 0.6 (95% CIs: 0.20–0.90). Finally, during the wet season, log body mass was the only predictor retained to describe significantly higher RAIs at trail placements, with larger species showing a higher probability than smaller ones (Fig 4).

## Discussion

Non-random camera trap placement in the context of multi-species surveys violates a key principle of sampling theory: the random selection of sampling units [10]. Although the influence of placement strategy has been investigated in the past, few studies have implemented designs that specifically controlled for spatial or temporal confounding factors [23,24,45], and none that we are aware of in savannah habitats. In our study, the random and trail-based surveys sampled the same locations at the same time, enabling us to test more rigorously the influence of placement strategy on the observed richness, composition and structure of a terrestrial mammal community. Although we did not have an exact reference community against which to compare our results, we were able to assess relative differences between community patterns resulting from random and trail-based camera trap placement strategies (Table 3).

**Table 3:** Advantages (pros) and disadvantages (cons) of random and game trail-based camera trap placement strategies in relation to different types of survey aims, based on a comparison carried out in Ruaha National Park, Tanzania.

Placement strategy	Community richness and composition		Species presence/Occupancy		Space/Habitat use	
	Pros	Cons	Pros	Cons	Pros	Cons
Random	Detection of species that never use trails	Slower to detect many species so greater effort needed to capture full community	More likely to detect species that never use trails	Less likely to detect larger-bodied and carnivore species	More landscape features sampled	May need more sampling effort to obtain adequate sample size
		Fewer species detected when dense vegetation			More accurate characterisation of species preferences across the wider landscape	
Game trail-based	Detection of most species more rapidly	Non-detection of species that never use trails	More likely to detect larger-bodied and carnivore species	Less likely to detect species that never use trails	Larger sample size for many species (particularly larger-bodied and carnivore species)	Inferences will be restricted to trails
			More likely to detect species when vegetation is dense			

Although choice of placement strategy did not seem to affect overall community structure, it did have a marginal effect on observed community richness and composition during the wet season and for lower levels of sampling effort. In particular, species known to occur at naturally low densities, such as the caracal and the African wild dog, tended to be detected only by the trail-based survey during the wet season, indicating that this placement strategy may be more preferable for species inventorying at times when vegetation density may be higher off trails. Despite this, neither of the placement strategies implemented in this study was able to record all 41 species detected overall, indicating that neither offered a completely optimal design for species inventorying. For instance, the klipspringer and the African wild dog were only detected by the trail-based survey while the ground pangolin only by the random one. Nevertheless, for studies that can implement extensive surveys (i.e. > 1400 camera trap days on average), random camera placements may eventually yield a more complete list of species, especially since game trails will also be sampled in proportion to their occurrence [17]. Conversely, if sampling periods are short or the number of cameras available limited, trail-based camera placements may facilitate detection of more species, more rapidly.

In general, the use of RAIs to determine species rankings within observed communities cannot be recommended owing to the uncertainty as to whether these are truly correlated with species density [31,46]. However, we were interested in assessing relative changes in species rankings between two surveys sampling the same locations at the same time. Our finding that species can show significant shifts in rank between two surveys adopting different placement strategies—reflecting relative changes in RAIs—supports previous conclusions [9,10,18]. For instance, Sollmann et al. [47] showed that species-specific response to different types of “trap setups” biased RAIs drawn from camera trap data. We found that RAIs could be significantly higher at trail placements depending on trophic category or body size of the species in question during the dry and wet season, respectively, thereby influencing observed rank in the corresponding community.

Carnivores, in particular, had significantly higher RAIs at trail placements during the dry season. Carnivore preference for trails is well known and has influenced the placement of camera traps since systematic surveys aimed at estimating abundance of rare and elusive felids were first implemented [13]. Despite this, the reasons underlying the preferential use of trails by carnivores remain less well understood, but causes may be grounded in optimal foraging theory, which dictates that individuals should attempt to maximise net energy gain per unit time, or minimise travel costs [48,49]. For species that defend a territory or whose resources are heterogeneously distributed in space, such as most carnivores and some insectivores (e.g. the aardwolf), trails may represent cost-effective patrolling routes or links between areas of high resource abundance. In the case of RNP’s large carnivores, the latter may correspond to areas close to the river where herbivores gather in the dry season.

In contrast, relative abundance indices for most herbivores did not differ between placement strategies. An important implication of this finding is that studies using trail placements so as to increase capture probabilities of carnivore species—in the context of mark-recapture analyses, for instance—may at the same time collect unbiased data on their potential prey. However, this may not be the case for all herbivore species, as shown by the significantly higher RAIs obtained for the giraffe, dikdik and hippopotamus at trail placements. Interestingly, while the former species may use trails as a consequence of its unique morphology, the second is known to be a territorial species whose use of trails may be linked to patrolling activities. Finally, hippopotamuses, which are primarily detected in proximity to the Great Ruaha River in the dry season, are known to follow trails between the water’s edge and nocturnal grazing grounds.

A number of reasons may explain the positive relationship between species body mass and significantly higher RAIs measured at trail placements during the wet season. Firstly, it may be more energetically costly for larger bodied species to travel through the denser vegetation typically found at this time of the year outside of trails. Secondly, larger bodied species often create and maintain the network of trails occurring in the landscape, and may therefore use them out of habit. Finally, the size of trails used by a species may be proportional to its body size. Smaller species may favour narrower trails than the ones considered in this study (≥ 1 m in width), which may be used preferentially by larger bodied animals.

## Conclusions

Our study has shown that, given adequate sampling effort (> 1400 camera trap nights), placement strategy is unlikely to affect inferences made at the community level. While differences in community richness were notable in the wet season and for lower levels of sampling effort, patterns of community composition and structure as revealed by random and game trail-based camera placements were similar overall. In contrast, and in agreement with previous work, placement strategy was found to influence capture rates of individual species, and especially those of carnivores and larger bodied species during the dry and wet season, respectively. Although our study was based in a relatively open east African landscape, our work should ideally be replicated in forested habitats, where, given the more cluttered nature of the habitat, it can be expected that differences in observed communities will be greater.

## Supporting Information

S1 Table Species-specific variables used in generalised linear models (GLMs) investigating the determinants of high relative abundance indices (RAIs) at trail placements. (DOCX)

S2 Table Species-specific location shift between random and trail-based camera placements during the dry season. (DOCX)

S3 Table Species-specific location shift between random and trail-based camera placements during the wet season. (DOCX)
