# Supplementary material for: Random versus Game Trail-Based Camera Trap Placement Strategy for Monitoring Terrestrial Mammal Communities
Source: PLoS One. 2015 May 7;10(5):e0126373. doi: 10.1371/journal.pone.0126373 (PMC4423779; doi:10.1371/journal.pone.0126373)
Supplement: S1 Table — (DOCX) [file pone.0126373.s001.docx]

S1 Table.

| Species | Trophic category^a^ | Social behaviour | Mass^b^ | Binomial response^c^ | |
| --- | --- | --- | --- | --- | --- |
|  |  |  |  | Dry season | Wet season |
| Aardvark | Insectivore | Solitary | 52.4 | 0 | 0 |
| Aardwolf | Insectivore | Solitary | 10.0 | 1 | 1 |
| African civet | Omnivore | Solitary | 12.0 | 1 | 1 |
| Baboon | Omnivore | Social | 15.0 | 0 | 1 |
| Banded mongoose | Insectivore | Social | 1.9 | 1 | 0 |
| Bat-eared fox | Insectivore | Social | 4.2 | 0 | 0 |
| Black backed jackal | Carnivore | Social | 8.5 | 1 | 1 |
| Bush duiker | Herbivore | Solitary | 19.5 | 0 | 0 |
| Common genet | Omnivore | Solitary | 2.0 | 1 | 0 |
| Dikdik | Herbivore | Social | 5.3 | 1 | 1 |
| Eland | Herbivore | Social | 570.0 | 0 | 0 |
| Elephant | Herbivore | Social | 3940.0 | 0 | 1 |
| Giraffe | Herbivore | Social | 900.0 | 1 | 1 |
| Greater kudu | Herbivore | Social | 214.0 | 0 | 0 |
| Hippopotamus | Herbivore | Social | 1418.0 | 1 | 1 |
| Honey badger | Carnivore | Solitary | 8.0 | 1 | 0 |
| Impala | Herbivore | Social | 52.5 | 0 | 0 |
| Leopard | Carnivore | Solitary | 55.0 | 1 | 1 |
| Lesser kudu | Herbivore | Social | 81.6 | 0 | 0 |
| Lion | Carnivore | Social | 161.5 | 1 | 1 |
| Porcupine | Omnivore | Social | 20.0 | 1 | 1 |
| Serval | Carnivore | Solitary | 11.5 | 1 | 0 |
| Slender mongoose | Carnivore | Solitary | 0.6 | 0 | 0 |
| Spotted hyena | Carnivore | Social | 63.0 | 1 | 1 |
| Vervet monkey | Omnivore | Social | 5.0 | 0 | 0 |
| Warthog | Herbivore | Social | 82.5 | 0 | 0 |
| Waterbuck | Herbivore | Social | 210.0 | 0 | 0 |
| White-tailed mongoose | Carnivore | Solitary | 3.5 | 1 | 0 |
| Wild cat | Carnivore | Solitary | 4.7 | 1 | 0 |
| Zebra | Herbivore | Social | 400.0 | 0 | 0 |

For each of 30 species, trophic category is given alongside social behaviour (solitary or social) and average body mass (in kg).

^a^Based on Kisling et al. 2014.

^b^Based on Smith et al. 2003.

^c^Species for which the RAI at trail placements was found to be significantly higher than that at random placements were given a score of 1 whilst others were given a score of 0. The latter were used as response variables in season-specific GLMs with binomial errors and logit link functions.
