# Supplementary material for: Random versus Game Trail-Based Camera Trap Placement Strategy for Monitoring Terrestrial Mammal Communities
Source: PLoS One. 2015 May 7;10(5):e0126373. doi: 10.1371/journal.pone.0126373 (PMC4423779; doi:10.1371/journal.pone.0126373)
Supplement: S3 Table — (DOCX) [file pone.0126373.s003.docx]

S3 Table. Wet season.

|  |  | 95% CI | |  |
| --- | --- | --- | --- | --- |
| Species | Location shift^a^ | Lower | Upper | P-value |
| Aardvark | -1.64 | -4.92 | 1.64 | 0.326 |
| Aardwolf | -2.46 | -4.10 | -0.82 | < 0.05 |
| African civet | -1.64 | -2.44 | -0.84 | < 0.05 |
| Baboon | -1.26 | -2.53 | -0.01 | < 0.05 |
| Banded mongoose | -1.14 | -3.28 | 1.00 | 0.272 |
| Bat-eared fox | 0.14 | -4.10 | 4.38 | 0.905 |
| Black backed jackal | -2.50 | -4.97 | -0.03 | < 0.05 |
| Bush duiker | -1.64 | -4.10 | 0.82 | 0.230 |
| Common genet | -0.82 | -2.46 | 0.82 | 0.246 |
| Dikdik | -5.45 | -9.26 | -1.64 | < 0.01 |
| Eland | 0.00 | -2.07 | 2.07 | 0.500 |
| Elephant | -14.87 | -28.75 | -1.00 | < 0.05 |
| Giraffe | -2.46 | -4.10 | -0.82 | < 0.01 |
| Greater kudu | -0.52 | -2.46 | 1.43 | 0.623 |
| Hippo | -7.50 | -12.54 | -2.46 | < 0.001 |
| Honey badger | 0.50 | -3.92 | 4.92 | 0.786 |
| Impala | -2.46 | -11.48 | 6.56 | 0.538 |
| Leopard | -1.72 | -2.52 | -0.92 | < 0.05 |
| Lesser kudu | 1.64 | -1.64 | 4.92 | 0.684 |
| Lion | -2.46 | -3.60 | -1.32 | < 0.01 |
| Porcupine | -1.65 | -2.46 | -0.85 | < 0.01 |
| Serval | 0.66 | -1.13 | 2.46 | 0.659 |
| Slender mongoose | -1.64 | -3.28 | 0.00 | 0.345 |
| Spotted hyena | -4.10 | -5.74 | -2.46 | < 0.001 |
| Vervet monkey | -2.94 | -10.80 | 4.92 | 0.416 |
| Warthog | -1.51 | -3.60 | 0.57 | 0.252 |
| Waterbuck | -1.64 | -3.28 | 0.00 | 0.345 |
| White tailed mongoose | -1.64 | -3.32 | 0.04 | 0.112 |
| Wildcat | 1.62 | 0.11 | 3.12 | 0.181 |
| Zebra | 0.64 | -2.97 | 4.24 | 0.706 |

Comparisons were based on pairwise Wilcoxon signed rank tests. These tested the null hypothesis that the distribution of pairwise differences (trapping rates at random placements minus those at trail placements) was symmetric about zero. The mean of the resulting normal distribution – termed the location shift – and the associated 95% confidence intervals were used to assess the level of significance (α = 0.05) relative to zero.
